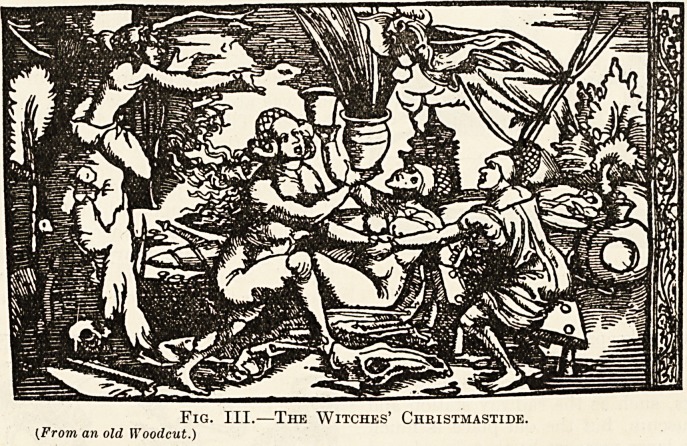# Christmas Appeal Number

**Published:** 1909-12-18

**Authors:** 


### CHRISTMAS APPEAL NUMBER.

### CHRISTMAS AND THE HOSPITALS.

At no other time of the year is the appeal which our charitable institutions make to the general public so strong as it is at Christmas; at no other time, it is permissible to suggest, should that appeal be considered more carefully, or be responded to more generously. With very few exceptions—so few that for all practical purposes they may be entirely disregarded—our hospitals exist upon the Voluntary System. That is to say, they depend for their upkeep entirely upon the willing charity and spontaneous generosity of individuals. They get no State aid, no loans or contributions from the rates. They owe their daily bread entirely to the munificence and generosity of private contributors, just as much as Barnardo's Homes do or any orphanage supported by voluntary contributions.

### What the Voluntary Principle Means.

For the moment it is worth pausing to consider what it means, this voluntary principle on which our hospitals are founded, and on which they have for centuries stood secure. How many of us can tell, if asked, what is the annual or the daily cost of upkeep of one of our large Metropolitan hospitals? The cost is relatively small when it is taken into consideration that a huge amount of work is done—work which could not otherwise be performed or done in any other place—but it is very large, and those to whom the hospitals look for support have but a small conception of what is spent and how it is spent. We have heard on more than one occasion the expression of opinion dropped from the lips of a reader of a hospital report that the hospitals waste money: it is an opinion which is grossly unfair to the majority of our institutions, and which does not take cognisance of the manifold expenses with which the management of a modern hospital, wishful of keeping its establishment up to date, has to cope. The daily bread of a large hospital means an outlay of many five-pound notes—in the case of a hospital like St. Thomas's or the London, it means hundreds of pounds, each farthing of which has to be accounted for. And, adhering as our institutions do to the voluntary principle, which in its essence is a principle of trust in the people, faith in the fundamental generosity of the public, complete confidence in the underlying streak of kindliness which each human being possesses, they have to get this money, so many hundreds of thousands a year, by appealing to the charitable and waiting for a response to that appeal.

### A Note on Hospital History.

In other countries, where State or Municipal aid is given to the hospitals, the case is entirely different. There the hospital is a State or municipal burden: its upkeep means a tax upon the shoulder of every citizen. Charity by taxation is hardly charity, and the adoption of the State system robs the hospital of one of its most honourable claims—that it is an institution where the sick poor are tended and cared for by the charitable. That was, at least, the old definition of a hospitalarium, such as the monks established in the first century A.D. That, too, was the definition applicable to the oldest hospitals of which we have any record—the hospitals of the times of Moses, the hospitals of Greece, India, and Assyria. The Egyptian hospital was not a State-aided institution; it was an establishment supported by the generosity of rich and poor alike. The rich man was attended by the hospital doctor—for there were specialists in those days, as anyone reading the excellent descriptions of Egyptian hospitals in Haeser's monumental work on medical history can see—and paid a donation when he recovered or left a legacy when he died. The poor woman brought payment in money or kind, not for services rendered by the institution itself, but for "favours granted by the Gods." Very much on the same lines were the monkish infirmaries or hospitals in the early days of the Christian Era. They were founded by kindly souled priests, chief among whom was the benevolent Bishop Basileus, who gave their whole lives, and in many cases their fortunes, for the relief of the sick, and who did their utmost to inculcate into their flocks the blessedness of giving alms and relieving the poor. Let us bear in mind that there is nothing new under the sun, and that Hospital Sunday and the voluntary system are as old as hospitals themselves, for Bishop Basileus preached his hospital sermon "with highest benignity," says the old chronicle, "and lissom-lipped in the cause of the destitute, the halt, the moribund, the deformed, the sick, and the suffering." He recognised the catholicity of pain and the offset against it—the universality of sympathy—and his sermon, which alas! has only come down to us in fragmentary form, eloquently shows how well he used his knowledge. Wise in his knowledge of human kind, he selected the approach of Christmas—not then, as now, a feast of jollification, merry making, and joyfulness so much as a day of solemn thanksgiving and fervent elation—for the preaching of his hospital sermons, and he kept up his hospital entirely upon voluntary contributions.

### The Great Text.

"Times have changed and manners with them." No doubt the often-quoted phrase is as true as it is trite, but with all our alterations which the revolving wheel of time has brought about we have not got rid of sickness nor eliminated suffering from our civilisation. Were there need at Christmas time to preach a sermon on the hospital appeal, this would be the text, and its truth would be too obvious to demand amplified demonstration. "The sick poor are always with us." Reform in the Poor Law, in our hospital system, in our methods of treatment —they are all very well and excellent in their way, but the present need is the great need, the support of the hospitals that look for their support to every member of the public, whether in sound health or sick. We have already said that hospital expenses are heavy; let us add that they are, and of necessity must be, increasing. The sick poor in a modern hospital are granted the benefits of the latest and most improved methods of treatment—methods which are always costly, which demand more men and more money. Take, for example, the *x*-rays. They are necessary both for what are called "diagnostic purposes"—that is, for finding out in obscure cases what is the matter with the patient—and for curing him in certain diseases. No modern hospital can be deemed complete without possessing a fully equipped *x*-ray department, which means a specially fitted photographer's workshop, with a specially trained staff, special costly and delicate apparatus, and a special annual expense in keeping all this up. The *x*-ray department is only one of the many special departments which a modern hospital has to establish. In addition there are the electric and Finsen light departments—or, to generalise them, the actino-therapeutic or light-healing department—improved laboratories for serum diagnosis and vaccine treatment, departments for special diseases (we are not now concerned with so-called research departments, but solely with those designed for the actual treatment and relief of patients), and many more. All these necessitate added expenses and call for extra help from voluntary helpers. Every year the struggle with debt is becoming greater; every year there is more help wanted.

### Help the Hospitals.

It is a supreme honour to the English public—an honour shared by no other community in the world to the same extent—that since the introduction of hospitals in this country the voluntary principle has worked so admirably, and found such staunch support outside the hospitals. We do not want to discuss the merits of the system here: we are only concerned with this simple fact, that it is the universal system in England so far as general hospitals are concerned. At this season it behoves everyone to have a thought for the hospitals. There is no question that the well are as interested in these institutions as the sick, the rich as much as the poor. We who are outside the walls enjoying the crispness of the December days, knowing

God's in His heaven!
All's right with the world!

—we at least should have a spare moment and a spare mite to contribute towards the alleviation of the suffering within. At any moment we, too, may claim the benefits so freely extended to them, but that should be the least of the thoughts of him who gives towards the hospitals at Christmas time. The charity that anticipates troubles to come is as immoral as the gratitude that is built upon prospective favours, and does not stand in need of Father Fuller's denunciation for its condemnation. Nor is it that kind of charity that will help the hospitals. The kind of hospital friend wanted, not only at Christmas time but all the year round, is the man or woman who will make him or herself acquainted with the needs of our institutions, who will spare a moment for personal service and interest others in the work, and who will strive to understand what these institutions are doing and how they are doing it. The greatest enemy of the hospital Christmas appeal is apathy and want of interest. Those who will endeavour to consider, be it ever so briefly, one of these numerous appeals, the nearest and closest for preference, which our hospitals are issuing at this season, will find that their appreciation of and their interest in the work that is being done for the sick poor will outlast many Christmases, and will give them many practical opportunities of showing that they are truly generous and charitable.

### AN OLD-TIME HOSPITAL CHRISTMAS.

One of the oldest of European hospitals is the fine old institution controlled by the Barmherzige Brüder in Prague. Standing on its old site, overlooked by the Hradshin towers and the Bohemian Mountains that encircle that most beautiful of cities, it nestles among a group of ancient buildings leading out of the Holy Ghost Lane. For several hundred years the brothers have lived here, generation after generation, and have tended the sick and given their hospital and its methods a reputation which few other similar institutions can boast of. The foundation of the institution dates back to the Middle Ages, when hospitals were haunts of misery and despair, dead houses almost, where the sick crept to die, where light and air were at a discount and good nursing was unknown. To the Charitable Brothers belongs the credit of having introduced order into the chaos that ruled in Austrian hospitals of a similar class, and to-day their hospital, while retaining its old-time quaintness, just as the Brothers have retained their old-time garb, their ancient manners, and their mild gentleness, may fairly claim to rank as a modern establishment. It has an up-to-date operating theatre and an ably qualified lay staff, but the nursing work, excellently done, is entirely in the hands of the Brethren.

It is interesting to glance in imagination back at the hospital Christmas in those early days when the Brethren began their good work in the wonderful city of Prague, wonderful then as much as now, for it is one of the most picturesque of old-world towns, brimful of colour and interest, and rich with an old-time glamour that pervades every street and alley way. In the cold, clear evenings that precede the Christ Birthday, Prague looks at its best. There is a flush of lilac behind the clouds, looming over the Hradshin, that old palace, half-pleasure house, half-fortress, and wholly a work of architectural art, making a gorgeous curtain for its turrets and pinnacled gables. The river winds slowly through the town, underneath that fine old bridge, guarded by its many statues of saints and holy men, and the old Powder Tower, a gigantic and

FIG. I.—A QUACK DOCTOR AT A CHRISTMAS FAIR.
*(From an Oldtime Wood Cut.)*

semi-solitary sentinel keeps watch over the lower town. Little has changed, perhaps, in the general outline of the city. There are new houses, new vast blocks of buildings, such as the railway station and the Bohemian Museum, but the old place is much the same, and in a large measure he that is endowed with any imagination can easily reconstruct much of the picture of four hundred years ago.

### THE OLD HOSPITAL.

In those days the hospital of the Brothers of Charity had the field much to itself. To-day there are many clinics at Prague and the brethren have competitors, not as many as they wish to see, for competition in hospital work is always to be welcomed, but sufficient to break the edge of the demand in their old city. Then it was different. Theirs was the only refuge for the sick, and the itinerant quacksalvers, who paraded in front of their house at Christmas time, only gave them more work and more patients. The quack doctor set up his booth at the fair, preferably in the vicinity of the hospital, and preferably, too, at New Year or Christmas, though the feast of St. Portiuncula later on became his day of election. The old-time medical books give him much attention, and in the archives of the order he is frequently referred to, not always slightingly, for there appear to have been quacksalvers who supported the hospitals and did much good to the poor. The ordinary quack dealt in teeth extractions, elixirs, magic powders, tape-worm salves, and other matters, and at Christmas time he was much patronised by the public.

The doors of the Brethren's hospital were opened wide on Christmas Eve, and in the large court-yard of the hospital was built a fire, huge, immense. Round it sat the brethren, headed by the Prior, whom one loves to think of as a man befitting in face and feature the roaring welcoming fire over which he presided—a stout, jolly-faced monk, kindly and benevolent. In the rear of the circle crowded whoever cared to join. Poor people came and sick people, the convalescents from the wards, and the old patients from afar off, and when the night was too boisterous, the party adjourned to the large hospital refectory. This was the Dole fire at which Christmas-boxes were given to the poor, primarily to the sick poor, just as to-day at many of our hospitals the present and old patients are welcomed at a Christmas out-patient tea. Gifts of food and clothing, gathered by the monks during the past year, were distributed by the Prior and the Almoners, and mead was served out and roast meat eaten. The ceremony lasted some time, but was never prolonged until after midnight, and was always concluded by a carol and the benediction. Then the party dispersed and the Brethren retired to the wards to watch over dying patients. For on Christmas Eve, as an old superstition alleges, many more sick people die than on any other night. "It is the night upon which God pleases," says the old chronicle, "to gather his lambs unto him—to give those that are weary, rest, those that are faint, food." So at least the popular theory ran, and in those days there were other theories about Christmas

FIG. II.—THE DEATH DANCE.
*(From an Old Copper Print.)*

Eve as well. Did not the dead from the hospital grave-yard swarm out of their graves when the Prior's party had retired, and annually dance round the smouldering remains of the Dole fire? Did not, when they had done their revels, the witches gather in groups and hold their meetings, invoking Master Leonard, who has

Goat's face with feet of kine, and drinking hippocras and the blood of unbaptised babes? That was evident enough, since more than one person, venturesome enough to watch the Dole fire after the retirement of the Brethren had seen them, and one or more artists had actually drawn them on the spot, as the wondering and curious may find in the collection of woodcuts in the old Prague Museum.

### Christmas Day.

The next morning there was bustle and excitement. On Christmas Day the wards were decorated. The high altar—for the Brethren keep altars in their wards, or did in those days—was brightly garnished with new flowers and burnished metal. Holly and mistletoe were fixed in the windows or strewn over the beds, and fresh rushes lay on the floor. The inside of a hospital ward in those days was very different from that of our modern institutions. There were few bedsteads. The sick were laid upon the floor, directly upon thin mattresses, and retained their clothing, or, where the hospitals could afford it, were dressed in a loose gown of sacking that was rarely washed. To modern eyes that Christmas ward must have seemed very horrible, very dreary, and very foul. But it was a change, and for the better, from the everyday condition of the ward, and the Brethren did their best to make the day pleasant for their patients. The Bishop, or sometimes even a Legate, the Count, or great patron of the hospital visited the institution on that day, and the patients crept round him, and probably his presence gave many of them encouragement. It is even on record that a King of Bohemia cured a paralysed man on his Christmas visit to a ward, and the influence of the Royal Touch for curing the King's Evil was, of course, a recognised belief in those days. Since the Brethren's Hospital belonged to a religious order, visits from high dignitaries of the church were frequent and doubtless much appreciated. Sometimes these visitors would bring a priceless relic for the hospital chapel. The power of faith in curing disease is very great, and even to-day, when the Brethren use vaccines, the newest surgery, and the most up-to-date methods, they do not scorn to turn to an old relic now and then, very often with the most beneficial results to their patients.

The visits over there came dinner. Ordinarily the fare provided for hospital patients in the Middle Ages was of the poorest. It consisted, as a general rule, of thin gruel and black bread, varied with occasional draughts of wine or beer. Meat was rarely served, and when dished up was flavourless and useless since all its value had been taken out of it by boiling it to obtain broth. On high days and festivals meat was served: very often game was sent by the patron, or bullocks meat, while the Brethren have never despised horse meat, which, as those who have tasted it know, is both succulent and of high nutritive value. On Christmas Day then the patients got beer and meat and turnips—potatoes not being common in Bohemia in those days—with some meal paste baked in oil and sweetened with honey as dessert. Then came afternoon service with a sermon. In the morning there had been early mass and high mass, and at night again there would be evening mass. Between the services the brothers who served as doctors went their rounds and dressed their cases, while the lay brothers tended the patients under their supervision.

Fig. III.—The Witches' Christmastide.
(*From an old Woodcut.*)

To-day the routine in the Brethren's Hospital is in some points very much the same, in others entirely different. The Dole fire is not kept up any longer; instead there is the refectory assembly and an out-patient dinner. The ghosts still dance out in the courtyard, though Socialism and modernism have made so much way in Bohemia that there are plenty of sceptics who refuse to believe that there are either witches or ghosts at Christmas or any other time. The hospital still retains its old fashions and its old-world quaintness, but it is a modern, excellently-equipped institution which no one who is interested in such matters and who visits Prague should lose the opportunity of visiting. The Brethren are hospitable and kind: they take a delight in showing visitors round, and their institution is well worth seeing.

### HOSPITALS WITH 150 BEDS AND UPWARDS.

### BIRMINGHAM GENERAL HOSPITAL.

THE applicants during the past year have been so numerous that, in spite of the more chronic patients being sent to the Jaffray Branch Hospital and to convalescent institutions, there has been a constant list of patients waiting for admission. 5,529 in- and 65,557 out-patients have found relief during the past year. Last year there was a deficit in the annual income of £10,060. In a busy manufacturing town like Birmingham there is an obvious need for a large efficient general hospital. Besides the ordinary population, the factory hands are numerous, notably poor, and their very occupation renders them liable to sickness and accident, while outside the actual district there is an immense population in the Midlands to be relieved in serious cases. If the increased work of the hospital is to be continued, more income is absolutely necessary, and an earnest appeal is made for new subscriptions and donations. House Governor, Mr. Howard J. Collins.

### BROMPTON HOSPITAL FOR CONSUMPTION.

THE first hospital of its kind to be built in this country, was established nearly 70 years ago, and contained 100 beds. Its history since then has been one of steady progress, and there is now accommodation for over 300 patients at Brompton, special wards for children, and also Jewish patients, a spacious out-patient department, where there are nearly 50,000 attendances annually, special departments for throat, ear, and nose cases, a dental department, Röntgen-ray department, and the clinical laboratories. At the Sanatorium at Frimley there are 150 beds, the greater number of which are used for patients from the hospital, the others having been added subsequently for the reception of patients able to pay the maintenance cost of 25s. per week, but who cannot afford the fees ordinarily charged at a sanatorium. The prizes awarded last year at the International Tuberculosis Congress at Washington to both the " Brompton " and " Frimley " exhibits demonstrate in a most striking manner not only the prominent position of the Brompton Hospital, but the leading part taken by Great Britain in the treatment of this terrible disease. Further financial support is greatly needed, and subscriptions and donations will be gratefully acknowledged by Mr. Frederick Wood, the Secretary.

### GUY'S HOSPITAL.

THE Hospital of Thomas Guy is at all times, as Mr. Gladstone declared some 14 years ago, performing a work of " elementary necessity," benefiting the individual sufferer and rendering not less important services to the public as a place of education and research. Upon these grounds the Governors base their claim to due and continued recognition of the needs of the charity. Last year no less than 8,565 in-patients were received, while the out-patients numbered 132,288. An earnest appeal is being made for contributions to meet the large annual deficiency between assured income and necessary expenditure, and funds are also required to provide children's wards and badly-needed increase of beds for special departments. Treasurer. Mr. H. Cosmo Bonsor, London Bridge, S.E.

### KING'S COLLEGE HOSPITAL.

VERY rapid progress has been made with the new King's College Hospital during the past year. The outside of the building is finished, and the scaffolding has been removed. Work on the central or administration block has also advanced, and before long the erection of the ward blocks will be proceeded with. On July 20 his Majesty the King was graciously pleased to lay the foundation stone of the new building. After congratulating the committee on their wise and right decision to remove the hospital, and on the happy result of their efforts, his Majesty concluded his speech with these words:—

> " I am glad that the fund associated with my name favoured the scheme in its inception, and has given material assistance in carrying it out; and I am confident that other generous donors will come forward to supply the money needed to complete a spacious and well-equipped building. I can conceive no worthier object for benevolence than this."

Additional contributions are required to meet the current expenses of the hospital. Secretary, Capt. H. S. Tunnard, Portugal Street, Lincoln's Inn Fields, W.C.

### LONDON HOSPITAL.

THIS hospital is situated in the East End, where its influence for good is not confined to the medical relief it affords, for the humanising and civilising influences which result to a patient, from a residence within its walls, are of immense value to the citizens. Its work improves the character of large numbers of the population throughout one of the poorest districts of London. This may also be said with truth in regard to other hospitals, but the influence in the case of the London is more patent and appreciable owing to the vast numbers handled every year. The ordinary income, including donations, subscriptions, etc., is not much over £80,000, and the cost of upkeep is over £100,000. It would be a calamity to East London if the work of this noble institution had to be curtailed. Secretary, Mr. E. W. Morris.

### MIDDLESEX HOSPITAL.

THE general wards of this hospital accommodate 305 patients, and all treatment is entirely free. The assured income of the institution is disproportionate to the necessary expenditure, and, in consequence, it is mainly dependent on voluntary contributions for its maintenance. A unique feature of the

Middlesex Hospital is its special Cancer Charity (forty-seven beds), where those stricken by this disease may obtain such treatment and relief from their sufferings as the highest medical skill, coupled with the sympathetic ministration of experienced nurses, can afford. The scientific investigation into the cause and cure of cancer has been systematically carried on since January 1900. These few facts serve to show that the Middlesex Hospital is eminently worthy of the liberal support of the public. Secretary-Superintendent, Mr. F. Clare Melhado, Mortimer Street, W.

### MOUNT VERNON HOSPITAL FOR CONSUMPTION AND DISEASES OF THE CHEST.

From small beginnings this hospital has grown to a great national charity. Its two hospitals contain 220 beds, all of which are occupied. In addition the out-patient department in Fitzroy Square is largely attended, and is doing a useful and most important work. Some idea of this work may be gathered from the fact that patients are received from all parts of the Kingdom, and unhappily many have to be turned away because funds are insufficient to provide for them. It is engaged in a perpetual war against consumption, and is educating the public in regard to the prevention, as well as the cure, of a disease which is a veritable scourge. At the present moment a sum of £3,000 is required to carry on the work and to avoid the incubus of debt. Apart from the relief which the hospital affords, its work is of the greatest value to the nation, and deserves the support of every person who would wish to see consumption prevented and stamped out. Contributions may be sent to the Secretary, Mr. W. J. Morton, at the offices, 7 Fitzroy Square, W.

### ROYAL FREE HOSPITAL.

This hospital was founded in 1828 on the principle of free and unrestricted admission of the Sick Poor; poverty and suffering being the only passports required. Having no endowment, it is entirely dependent on voluntary subscriptions, donations, and bequests. Over 2,500 poor sick persons are admitted to the wards annually, and advice and medicine is administered to about 40,000 out-patients and casualty cases, who resort to it, not only from the crowded courts and alleys in its neighbourhood, but from all parts of London and the suburban districts. The relief thus afforded costs about £16,000 per annum, while the reliable income does not exceed one-third of that sum. Moreover, it has been needful during the past few years to expend large sums upon various structural and other improvements, which have been necessary in order to bring the hospital up to the standard of modern requirements. With this heavy additional outlay the committee have been obliged to make strenuous efforts to avoid getting into debt, and they earnestly solicit increased support from the charitable public, so as to enable them to carry on the beneficent work of the hospital in an efficient manner. Contributions will be thankfully received by the Treasurers, or by the Secretary, Mr. Conrad W. Thies, Gray's Inn Road, W.C.

### ST. GEORGE'S HOSPITAL.

It is thought that many people who might subscribe to this charity refrain from doing so from the impression that, as it is situated in a wealthy neighbourhood, it is necessarily a rich hospital. The ordinary expenditure exceeded the ordinary income in 1908 by £18,917. To meet this the hospital was compelled to sell out stock and use legacies. No wards are closed, but the whole of the beds are used to the best advantage for the public. Some years back the annual subscriptions amounted to over £8,200, whereas in 1908 they had fallen to £5,507. The number of new in-patients treated in 1908 was 4,389, and out-patients 41,934. St. George's has the largest convalescent hospital attached to any individual hospital in England, containing 100 beds, to which 1,300 of the in-patients were sent in 1908 without charge. The hospital has a most complete electrical department, fitted with the Finsen light and *x*-ray apparatus, and everything possible that scientific research can accomplish is done for the alleviation of the sick. Secretary to House Committee, Mr. H. Wingrove, Hyde Park Corner, S.W.

### ST. MARY'S HOSPITAL, PADDINGTON.

Few institutions can possess a more powerful claim to public support than that of the general hospital of Paddington. Whether that claim be based on the past record of the hospital, its present work, or its promise for the future, it is impossible to deny its force. Of St. Mary's past the *Times* in a recent article said: "It has had no time to build up venerable traditions, but from the day of its opening (1851) it has continued to hold a high place among its contemporaries and its reputation has been continually enhanced." Concerning the future, may not St. Mary's be said to be the birthplace and nursery of treatment by therapeutic inoculation, the great possibilities of which are now receiving universal recognition? But, in spite of all these claims, the hospital is inadequately supported. Last year ended with a deficit of £2,400, and there is reason to fear that this year will close with a still larger debt. If this fear be realised, the closing of beds cannot be far off, for there is no funded property to speak of. The public should see to it that this calamity does not befall the poor of Paddington and the surrounding district. Secretary, Mr. Thomas Ryan.

### SEAMEN'S HOSPITAL SOCIETY (DREADNOUGHT).

Few of the general public give a thought to sailors, and yet they suffer more than any other class of the community from accident and injury, while everyone in these sea-girt isles owes a deep debt of gratitude to them. While workers on shore need our sympathy when ill, how much more do those who toil on the sea? They work seven days in the week, they are exposed to the ravages of the diseases of tropical climates, and when they are stricken down by accident or illness it is often on the high seas without the succour of doctor or nurse. The Seamen's Hospital, or as it is more popularly known, the "Dreadnought," receives these makers of Empire who travel to it from every port in the kingdom and even from abroad, and it is on behalf of these men in the hour of their trial that the Seamen's Hospital Society appeals to the public to at any rate give some portion of their benevolence to the sailor. It is on the ground that he is out of sight and unable to appeal for himself that the committee beg on his behalf for a share of your generosity. Over 30,000 sick and injured seamen are treated in the hospitals and dispensaries of the Society every year. The committee solicit bequests, donations, and annual subscriptions. Contributions may be sent direct to the Secretary, Mr. P. Michelli, C.M.G., at the "Dreadnought" Hospital, Greenwich.

### THE NATIONAL HOSPITAL FOR THE PARALYSED AND EPILEPTIC.

There is such a demand for in-patient treatment at this hospital that over 120 patients are on the waiting list. These are all carefully chosen cases, and it is probable that many of them will die or get worse before their time comes for admission. Unhappily, while there is difficulty in maintaining the present 160 beds, nothing can be done to ameliorate the lot of these poor people, but in all other directions the hospital has made great strides. In consequence of the success of the Duchess of Albany's Jubilee Fund, the out-patient department, the electrical rooms, the nurses' quarters, and many other important parts of the institution have been greatly improved. It may be said that provision has been made for the more important action of dealing with the problem of making further provision for in-patients. What is chiefly needed in the meantime is more annual subscriptions, and these are earnestly appealed for by the Secretary, Mr. Godfrey H. Hamilton, Queen Square, Bloomsbury, W.C.

### UNIVERSITY COLLEGE HOSPITAL.

At this season, when appeals for charity are numerous, the committee earnestly trust that the indisputable claims of University College Hospital to a larger measure of support than it has hitherto received will be recognised, and in this connection would specially draw attention to the fact that the list of subscribers is probably the smallest in the Metropolis, having regard to the size and importance of the institution and the wide extent of its work. The new hospital building, erected at a cost of over £200,000 by the late Sir J. Blundell Maple, Bart., M.P., whilst affording special and unrivalled facilities for the treatment of the sick, has imposed upon the committee the additional responsibility of meeting an increased expenditure for maintenance of £9,000. There is consequently a deficit of about £8,000, to meet which this appeal is being made. The committee trust that this appeal may meet with a truly liberal response, which will assist them in carrying out to its fullest extent their work of charity. They also venture to urge the claims of the charity upon testators. Treasurer and Chairman, Mr. Henry Lucas, Gower Street, W.C.

### WESTMINSTER HOSPITAL.

This is the oldest general hospital in London dependent upon voluntary support, and is carrying on a most necessary and important work in providing relief in a poor and congested district. For many years its ordinary expenditure has unfortunately been far in excess of its assured income, and at present the amount due to the bankers for overdraft is not less than £3,800. Last year 2,489 in-patients were received, while the number of out-patients reached 20,801. The committee make an urgent appeal in order to meet the demands upon this hospital. Nearly £20,000 has to be found every year, and it is essential that there should be a material increase in annual subscribers and donors if the relief afforded by this hospital is to be continued. Secretary, Mr. Sidney M. Quennell, Broad Sanctuary, S.W.

### Hospitals with Under 150 Beds.

### CANCER HOSPITAL (FREE).

This hospital is the only special hospital in London for the treatment of cancer. This treatment involves great expense, for not only have dressings and drugs to be supplied in liberal quantities, but the patients often need a generous and expensive dietary. It will thus be understood what a boon such a special hospital as the Cancer Hospital is to the poor, where they will receive not only the best medical and surgical treatment, but will also be nursed and fed in a manner far above their means to procure. The maintenance of such an institution is therefore necessarily most expensive, and the committee of management earnestly appeal for support in their endeavours to carry on the good work. The hospital is quite free, neither letters of recommendation nor payment being necessary, the only passports required being that the applicants shall be in necessitous circumstances and suffering from cancer, tumours, or allied diseases. Steps are being taken to erect within the grounds of the hospital a separate building as a research department, where laboratory accommodation will be provided for the separate branches of medical science in which the research of cancer should be pursued. This will add greatly to the expenditure, but the committee feel that work of such world-wide importance will receive adequate support. Secretary, Mr. Fred. W. Howell, Fulham Road, London, S.W.

### GREAT NORTHERN CENTRAL HOSPITAL.

This is one of the most modern of London's hospitals, the present building having been constructed in 1894. It has 167 beds, and over 2,300 in-patients and 27,000 out-patients are relieved annually. The hospital has risen in a comparatively few years from a small beginning, and is now the largest of the general hospitals without medical schools. Of the £16,000 required annually to maintain it, only £6,000 can be relied upon. The hospital has this year experienced a very serious and quite unprecedented falling off of income, the receipts being £7,000 less than the outgoings. At present over £12,000 is owing to the bankers for advances on current account, and with Christmas bills to meet the financial outlook is serious. The funds of this hospital are carefully and economically administered; it is doing excellent work in a very poor district and deserves a larger measure of support. Secretary, Mr. L. H. Glenton-Kerr, Holloway Road, N.

### HAMPSTEAD GENERAL HOSPITAL (with which is amalgamated the NORTH-WEST LONDON HOSPITAL).

The work, both at Hampstead, where 115 beds are now provided, and in the out-patients' department in Kentish Town Road, proves the value of the hospital as one of the outer ring of metropolitan institutions. Considerable advantages have accrued to the poor of these and the more outlying northwestern districts through the amalgamation, as the surroundings of the Hampstead Hospital are specially favourable, and by the retention of the outpatients' department in Kentish Town, where the whole of the benefits previously afforded by the North-West London Hospital are continued. The hospital has contributory beds in which local medical men attend their own patients. The development of the hospital has been so rapid as to have involved an expenditure for maintenance much in excess of the reliable income, and the Council plead most earnestly for help to provide additional income to the exent of £5,000 per annum, and to meet an accumulated deficit of £4,000. Secretary, Mr. George Watts, Haverstock Hill, N.W.

### METROPOLITAN HOSPITAL.

This institution is situated in one of the poorest and most crowded districts of London, and is therefore most suitably placed for its purpose; but in consequence of its being far removed from the neighbourhood of the well-to-do, it unfortunately does not receive the support it so much needs and so well deserves. The Hospital has just been reopened for the reception of patients after having been closed for over four months. During this period extensive alterations, improvements, and much-needed repairs have been carried out, which were rendered necessary to bring the institution up to the standard of modern requirements. A special appeal is being made to cover the cost of these extensive and most necessary alterations. Secretary, Mr. J. C. Buchanan, at Kingsland Road, N.E.

### POPLAR HOSPITAL FOR ACCIDENTS.

Situated among a teeming population of poor hard-working people in a district which may be called the "workshop" as well as the "port" of London, this hospital is doing an excellent work. The demands on the institution have of late years greatly increased, and consequently further support is very necessary, especially as the Convalescent Home at Walton-on-the-Naze costs about £900 per annum in maintenance. Secretary, Mr. Percy Rogers.

### PRINCE OF WALES'S GENERAL HOSPITAL, TOTTENHAM.

The situation of this hospital is peculiar to itself. Tottenham has been described as the home of the unskilled, and one of the dormitories of London, where the dwellings of the poor appear to be hidden behind respectable houses, and, by thus escaping general observation, give rise to the impression that the district is capable of self-maintenance. As a matter of fact, there is little money there, and none for charity; moreover, prosperous business men have no occasion to go there as in the case of a manufacturing or shipping district. The result is that the hospital, which is dependent upon the help of the wealthy and benevolent, is very inadequately supported, and at the present is in debt to the extent of £7,000. Director, Mr. Frederick W. Drewett.

### THE QUEEN'S HOSPITAL FOR CHILDREN

(Late North-Eastern).

This hospital is in a particularly precarious financial position at the present time, so much so that it is feared that 62 of the beds will have to be closed at the end of the year if substantial assistance is not forthcoming meanwhile. What a calamity this would be may be realised when the reader is made aware that the numbers of patients this year are thousands ahead of any previous year. Great care is taken to prevent abuse of this charity, and the result of the committee's constant efforts in the direction of economy is shown by the fact that the weekly cost per in-patient last year was only 26*s*. 6*d*. The hospital has scarcely any endowment, and depends on voluntary contributions to the extent of £10,000 a year. It is extremely important that prompt assistance should be forthcoming to meet the present pressing needs. Secretary, Mr. T. Glenton-Kerr, at the Hospital, Hackney Road, E.

### ROYAL LONDON OPHTHALMIC HOSPITAL.

Last year this hospital, formerly known as Moorfields Eye Hospital, relieved 2,331 in-patients and 46,744 out-patients, while the number of attendances of out-patients was 120,366. A large proportion of the patients are women and children.

The expenditure for this year has not been met by the income, in spite of the efforts of the committee to keep the expenditure as low as efficiency will permit, and in spite of the contributions of patients, who are all asked to give what they can to the donation box. Twenty beds are closed for want of funds. The hospital owes £2,500 to its bankers and £2,600 to trade creditors. The committee have no hope of carrying on the full work of the hospital without generous and regular additional help. Patients from every part of London come to this hospital for relief, and the committee appeal to the charitable public in London and the Provinces to help the hospital in its hour of need. Secretary, Mr. Robert J. Bland, City Road, E.C.

### Hospitals with Under 100 Beds.

### CHELSEA HOSPITAL FOR WOMEN, FULHAM ROAD, S.W.

In order to make practicable the growing work of this Hospital, especially that of an operative nature, and to keep the hospital fully abreast of the latest improvements, considerable extra expense has had to be incurred. There is no diminution in the success of its work. Cases of the severest type coming within its scope are increasingly numerous, but the rate of mortality remains at the low figure of 1½ per cent. Nor are the results limited to happy discharge from the hospital. The convalescent home completes the good work and implants the health and strength so necessary for resumption of home or other duties. The benefits of this hospital are available for poor gentlewomen as well as for those of the poorest classes. All alike are treated with the consideration and privacy which their necessities demand. The shortage of funds above mentioned must necessitate some curtailment of this beneficent work unless help is forthcoming. The avoidance of such an unhappy condition depends not only on the continued support of all the old friends of the hospital, but also on a considerable number of new sympathisers being forthcoming. Annual subscriptions and other contributions will be most thankfully received by the Treasurer, Mr. Henry E. Wright, or by the Secretary, Mr. Herbert H. Jennings, at the Hospital, Fulham Road, S.W.

### QUEEN CHARLOTTE'S LYING-IN HOSPITAL.

During the 157 years of this hospital's existence upwards of 130,000 poor women have been attended either in the hospital or in their own homes. Last year 1,865 patients were delivered in the hospital and 2,169 in their own homes. The income during the past three years has, however, fallen far short of the expenditure. In 1907 there was a deficit of £673; in 1908 one of £1,044; and it is feared that the deficit for the current year will exceed £1,000. The need of help is, therefore, very great, and the committee earnestly appeal for liberal contributions, as the invested property of the hospital is very small, yielding about £600 per annum only. Subscriptions and donations may be sent to Messrs. Cocks, Biddulph and Co., 43 Charing Cross, S.W., or to the Secretary, Mr. Arthur Watts, at the Hospital, Marylebone Road, N.W.

### Hospitals with Less than 50 Beds.

### CITY OF LONDON LYING-IN HOSPITAL

The committee make an urgent appeal for funds to liquidate the heavy debt incurred by the compulsory rebuilding of the hospital. The rebuilding and furnishing have entailed an expenditure of £40,000, and, in order to partially meet this, the committee have been obliged to obtain a loan of £17,000 from the bankers. The hospital is situate in one of the very poorest parts of the Metropolis, its patients being mainly drawn from the vast working-class population of Bethnal Green, Hoxton, Shoreditch, St. Luke's, and Islington. It has carried on this great work of mercy for the past 157 years. Some 3,500 poor women are delivered annually. A donation of £1,000 will name a ward; a donation of £100 will name a bed; life governor's qualification, £10 10s. It must be a source of gratification to those who are able to pay for their children being born in surroundings of happiness and comfort to know that by giving a donation to this hospital they are able to extend similar blessings to their less fortunate sisters. Secretary, Mr. R. A. Owthwaite, City Road, E.C.

### HOSPITAL FOR EPILEPSY AND PARALYSIS.

Patients are received into this hospital, suffering from all kinds of nervous diseases, the great majority of the sufferers being inadmissible to general hospitals. It affords free treatment to the necessitous poor, but encourages all who are able, to pay what they can afford, and thus promotes providence and minimises abuses. Its beds are constantly occupied and the attendances of out-patients average 13,000 per annum. The maintenance of the hospital for the current year will cost about £3,500, and as £2,800 has been received in income the sum of £700 is still required to avoid a deficit. Secretary, Mr. H. W. Burleigh, 4 Maida Vale, W.

### GENERAL CHARITIES.

### THE CHURCH ARMY.

THE Church Army plays a great part in the life of the Church and country. It does work which no other organisation undertakes, and directs its energies particularly to helping the unemployed and outcast, for which purpose it requires liberal support. It stands in need of additional monetary assistance at this season in order that it may provide Christmas dinners for starving families. Contributions should be sent to Prebendary Carlile, Hon. Chief Secretary, 55 Bryanston Street, W.

### FREE CONVALESCENT HOMES.

ANYONE visiting the wards of a hospital, and seeing the weary-stricken faces lying there, would realise the necessity of a thorough change of air and scene to completely restore health. Since 1840 the Metropolitan Institution has, by providing free convalescent homes, been administering to this necessity. It now comprises four homes containing a total of 559 beds, and receiving annually over 7,500 patients. About £14,000 is needed yearly to maintain these homes, to which patients are admitted entirely free of charge upon the recommendation of subscribers, and nearly the entire amount has to be obtained from voluntary sources. In addition, £7,000 is required for the completion of the new home for men only, at Little Common, which was opened in 1905. For this sum 38 additional beds, which are much needed, could be provided. To free the institution from these burdens an increased number of donors and subscribers is needed. Secretary, Mr. Alex. Hayes, 32 Sackville Street, W.

### ROYAL ASSOCIATION IN AID OF THE DEAF AND DUMB.

THE committee of this Association solicit contributions to enable them to give the usual Christmas gifts to the deaf and dumb poor. Last year on Christmas Eve 381 of these poor afflicted people were supplied either with provisions or gifts of money, and on Christmas Day 45 men and youths were supplied with a substantial dinner. A large number were entertained to a hearty tea and an evening's amusement at meetings held in the different districts. Any balance that remains after the Christmas gifts have been provided for will be transferred to the general fund. To encourage provident habits among the deaf and dumb a penny bank has been established, and help is solicited to enable the committee to give the promised interest. Besides these special objects, the committee are constantly giving pecuniary assistance to the deaf and dumb in sickness and distress. During the past year 3,635 visits have been paid to the deaf and dumb and 4,652 on their behalf. Employment has been found for 145. There are 22,000 deaf and dumb persons in the United Kingdom, about 3,000 of whom reside in London, forming an isolated community incapable to a great extent of entering into the ordinary social relationships of life, or to plead on its own behalf. Help from those who enjoy the invaluable gifts of hearing and speech is requested for the Association and its work. Secretary, Mr. Thomas Cole, 419 Oxford Street, London, W.

### NATIONAL CHILDREN'S HOME AND ORPHANAGE.

THE annual report of this home shows how steadily and rapidly this institution has increased within the last few years. There are now more than 2,200 children in its 13 branches, 11 of which are in England, one in the Isle of Man, one in Canada. A special feature in the work of the National Children's Home is that its ministry is not confined to one class of child. A large proportion of the inmates are what would be called waifs and strays, but it also makes special provision for the orphan children of Christian parents, and in its Certified Industrial Branch deals with boys committed by magistrates. The passing of the Children Act has led to a considerable increase in the number of applications, and the children received within the last few months are as needy as any rescued in the last forty years. The ordinary income of the home is about £50,000 per annum. The total income, including special gifts, amounted to £65,000. Early in 1910 the Committee will open at Harpenden, Herts, a well-equipped sanatorium for children threatened with or in the early stages of consumption. A special appeal is now being made to clear the waiting list of 100 children before Christmas. For every five guineas specially contributed the Committee will make provision for one of these. Principal, Rev. Dr. Gregory, N.C.H.O., Bonner Road, N.E.

### LONDON ORPHAN ASYLUM, WATFORD.

ORPHANED children of the necessitous middle class are eligible for admission to this institution, and are assured of a Christian training and a sound commercial education which will enable them to fill responsible situations in after life. Seven thousand children have been so benefited since the charity was established. For nearly a century the institution has carried on its beneficent work, hopefully and reliantly maintaining its family of 500 boys and girls. Each year commences with a liability of £14,000 required from voluntary sources, and during the year 1909 the managers have had to borrow a very considerable sum. They appeal, therefore, for help, and contributions will be gratefully received by the Secretary, Mr. Henry C. Armiger, at the office, 3 Crosby Square, E.C.

### THE MARY WARDELL CONVALESCENT HOME FOR SCARLET FEVER.

THE valuable work which this home is performing is not realised, or it would receive a larger measure of support. Were it not for this institution there would be no place to which to send convalescents from scarlet fever. It has benefited 5,000 patients. Though originally intended for the working classes, it also admits patients from the upper classes. The fees paid are not nearly sufficient to maintain the home. A debt of £400 has been incurred in carrying out repairs which have been postponed for ten years through lack of funds, and there still remains to be paid off a loan of £200. The committee now plead for help, and especially solicit annual subscriptions. Miss Mary Wardell, Hon. Secretary, Stanmore, Middlesex.

## Figures and Tables

**Fig. I. f1:**
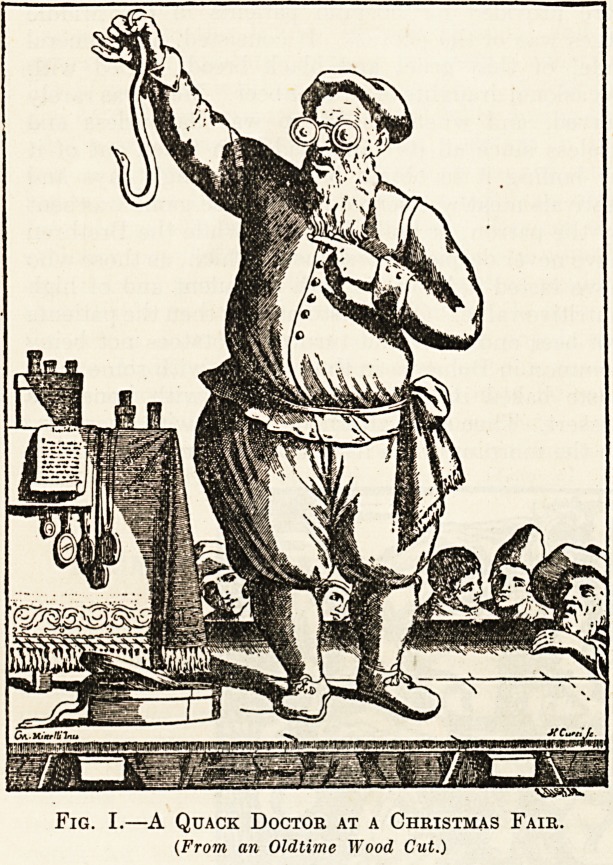


**Fig. II. f2:**
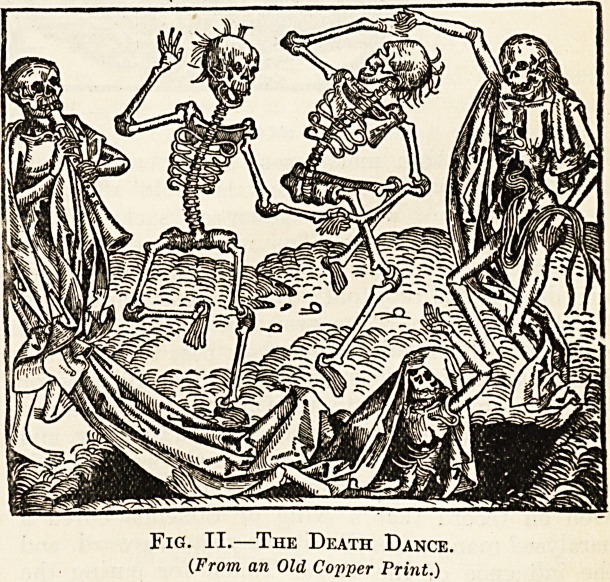


**Fig. III. f3:**